# Pre- and Postnatal Fine Particulate Matter Exposure and Childhood Cognitive and Adaptive Function

**DOI:** 10.3390/ijerph19073748

**Published:** 2022-03-22

**Authors:** Laura A. McGuinn, Lisa D. Wiggins, Heather E. Volk, Qian Di, Eric J. Moody, Eric Kasten, Joel Schwartz, Robert O. Wright, Laura A. Schieve, Gayle C. Windham, Julie L. Daniels

**Affiliations:** 1 Department of Environmental Medicine and Public Health, Icahn School of Medicine at Mount Sinai, New York, NY 10029, USA; robert.wright@mssm.edu; 2 National Center on Birth Defects and Developmental Disabilities, Centers for Disease Control and Prevention, Atlanta, GA 30333, USA; lsw0@cdc.gov (L.D.W.); ljs9@cdc.gov (L.A.S.); 3 Department of Mental Health, Johns Hopkins Bloomberg School of Public Health, Baltimore, MD 21205, USA; hvolk1@jhu.edu; 4 Vanke School of Public Health, Tsinghua University, Beijing 100084, China; qiandi@tsinghua.edu.cn; 5 Wyoming Institute of Disabilities, University of Wyoming, Laramie, WY 82071, USA; eric.moody@uwyo.edu; 6 Clinical & Translational Sciences Institute, Michigan State University, East Lansing, MI 48824, USA; kasten@msu.edu; 7 Department of Environmental Health, Harvard T.H. Chan School of Public Health, Boston, MA 02115, USA; jschwrtz@hsph.harvard.edu; 8 California Department of Public Health, Richmond, CA 94804, USA; gayle.windham@cdph.ca.gov; 9 Department of Epidemiology, Gillings School of Global Public Health, University of North Carolina at Chapel Hill, Chapel Hill, NC 27599, USA; julie_daniels@unc.edu

**Keywords:** air pollution, autism, cognitive functioning

## Abstract

Increasing evidence exists for an association between early life fine particulate matter (PM_2.5_) exposure and several neurodevelopmental outcomes, including autism spectrum disorder (ASD); however, the association between PM_2.5_ and adaptive and cognitive function remains poorly understood. Participants included 658 children with ASD, 771 with a non-ASD developmental disorder, and 849 population controls from the Study to Explore Early Development. Adaptive functioning was assessed in ASD cases using the Vineland Adaptive Behavior Scales (VABS); cognitive functioning was assessed in all groups using the Mullen Scales of Early Learning (MSEL). A satellite-based model was used to assign PM_2.5_ exposure averages during pregnancy, each trimester, and the first year of life. Linear regression was used to estimate beta coefficients and 95% confidence intervals, adjusting for maternal age, education, prenatal tobacco use, race-ethnicity, study site, and season of birth. PM_2.5_ exposure was associated with poorer VABS scores for several domains, including daily living skills and socialization. Associations were present between prenatal PM_2.5_ and lower MSEL scores for all groups combined; results were most prominent for population controls in stratified analyses. These data suggest that early life PM_2.5_ exposure is associated with specific aspects of cognitive and adaptive functioning in children with and without ASD.

## 1. Introduction

Autism spectrum disorder (ASD) is a group of neurodevelopmental disorders that affect communication, behavior, and social functioning. ASD can be reliably diagnosed by age two, though symptoms can appear as early as infancy [1]. Symptoms and severity of ASD vary widely from person to person; however, individuals with ASD often have impairments in adaptive behavior [2]. The term “adaptive behavior” refers to practical, everyday life skills needed to meet the demands of one’s environment [3]. Although adaptive behavior often tracks with age and IQ in typically developing individuals, for individuals with ASD and no intellectual disability, there is often a gap between cognitive ability and adaptive skills [4,5].

The etiology of ASD is poorly understood but likely includes contributions from both genetic and environmental factors [6,7,8]. Air pollution is one environmental factor that has been associated with ASD in a meta-analysis of recent epidemiologic studies [9]. Fine particulate matter (PM_2.5_) is a ubiquitous air pollutant and has been most consistently associated with ASD when exposure occurs during both the prenatal and early postnatal time periods [10,11,12,13]. Using data from the Study to Explore Early Development (SEED), we recently identified associations between late prenatal and first year of life PM_2.5_ exposure and the occurrence of ASD in children [14]. 

Studies have investigated the association between early life air pollution exposure and both ASD occurrence and severity of ASD symptoms, yet the potential impact of air pollution exposure on cognitive and adaptive function in children with and without ASD remains poorly understood [15]. Studying the impacts of environmental exposures on continuous cognitive and adaptive symptoms in children allows for the assessment of subclinical impacts and uses a more dimensional approach [16]. A few recent studies have found associations between prenatal and early postnatal air pollution exposure and several continuous cognitive outcomes in children in the general population, particularly for communicative domains, motor skills, and overall cognitive deficits [17,18,19]. Several additional questions remain including the specific window of susceptibility and whether associations are similar for children with and without ASD or a developmental delay (DD).

Building from our previous work in SEED, we first aim to assess the association between pre- and postnatal PM_2.5_ exposure and adaptive functioning among children with ASD. We additionally assess the associations of PM_2.5_ exposure with cognitive functioning in children with ASD, as well as children with developmental delays and population controls.

## 2. Materials and Methods 

### 2.1. Study Population

SEED is a multi-site, case-control study with study sites located in California, Colorado, Georgia, Maryland, North Carolina, and Pennsylvania. Children were eligible to participate in the first phase of SEED if they were born in a study site catchment area from 1 September 2003, through 31 August 2006, and still resided there at 30–68 months of age [20]. Eligibility criteria additionally included the availability of a knowledgeable caregiver to participate in English or Spanish (California and Colorado only). Three groups of children were invited to participate in SEED: (1) children with a diagnosis of ASD, ascertained through multiple sources that serve or evaluate children with developmental problems, including early intervention programs, special education programs, clinics, and individual providers; (2) children with developmental delays other than ASD (DD), identified from the same education and healthcare sources; and (3) population controls (POP), identified by randomly sampling state birth records of children born in the specified date range to mothers who resided in the study catchment areas at the time of delivery and still resided there at 30–68 months of age. 

Institutional review boards at each study site and at the CDC approved SEED (IRB #05-2660; approved September 2021). Informed consent was obtained from all enrolled participants. 

### 2.2. Outcome Ascertainment

Caregivers of all children completed the Social Communication Questionnaire (SCQ) [21]. Any child who screened positive on the SCQ, i.e., an SCQ score of ≥11, or reported a previous ASD diagnosis received a comprehensive in-person developmental assessment to determine final ASD classification. This assessment included two gold standard instruments, the Autism Diagnostic Observation Schedule (ADOS) [22] and the Autism Diagnostic Interview-Revised (ADI-R) [23,24]. Final ASD case classification was based on the results from the ADOS and ADI-R. 

The Vineland Adaptive Behavior Scale (VABS-II) was administered to parents of children who met the SEED criteria for ASD classification during the comprehensive assessment. The VABS is a semi-structured interview that assesses each child’s adaptive functioning compared to the functioning of others their age. VABS assesses adaptive behavior in several different domains, including communication, daily living skills, motor skills, and socialization. These four domains were summarized into the overall adaptive behavior composite score. VABS domain and composite scores are reported as standardized scores with a mean of 100 and standard deviation (SD) of 15. Lower VABS scores indicate greater impairment.

For all children (ASD, DD, POP), cognitive functioning was assessed using the Mullen Scales of Early Learning (MSEL) administered by trained clinicians [25]. The MSEL consists of an early learning composite (ELC) derived from four subscales: receptive language, expressive language, fine motor skills, and visual reception. MSEL ELC scores are reported as standard scores with a mean of 100 and SD of 15. MSEL subscale scores are reported as t-scores and age equivalent scores. Because many children from the ASD and DD groups received the minimum t-score on MSEL subscales (i.e., 20), we converted these scores to developmental quotients (DQ) by dividing the age-equivalent MSEL-score by each child’s chronological age and then multiplied this number by 100 [26]. Lower MSEL scores indicate greater impairment.

### 2.3. Exposure Assessment

The study participants’ addresses at birth were identified using birth certificates and matched to the nearest 1 km grid cell. The start date of pregnancy for each woman was calculated by subtracting the child’s gestational age (identified from the birth certificate) from their date of birth. To ensure participant’s privacy, dates of birth were randomly shifted by 0–14 days in either direction in the pooled data set. We previously assessed the extent of exposure misclassification for different exposure averaging periods and found larger misclassification for shorter averaging periods (weeks), but exposure misclassification was reduced for longer averaging periods (i.e., trimesters). Daily PM_2.5_ concentrations were averaged for the year post-birth, each trimester of pregnancy, including the first (weeks 1–13 of pregnancy), second (weeks 14–26), and third trimesters (weeks 27 to birth), and the entire pregnancy period.

Residential exposure to PM_2.5_ was estimated using a satellite-based exposure model [27]. Briefly, the prediction model incorporated data from a chemical transport model (GEOS-Chem); GEOS-Chem predictions were calibrated using monitored data. Land-use terms (percentage of urban areas, population density, road density, and elevation), meteorological variables (air temperature, precipitation, and wind speed), and satellite data were used to calibrate GEOS-Chem outputs and to aid in downscaling. This model additionally used a neural network to calibrate the predictors to monitored PM_2.5_ and was trained and validated with ten-fold cross-validation. The average PM_2.5_ concentration estimates were derived at a daily temporal resolution and a 1 × 1 km spatial resolution.

### 2.4. Statistical Analyses 

Bivariate analyses assessed differences in demographic characteristics and exposure levels by case status using analysis of variance and independent *t*-tests for continuous variables and chi-square tests for categorical variables (*p* < 0.05 was considered statistically significant). We assessed the distribution of air pollution levels across trimesters and calculated Spearman correlations comparing the levels across trimesters. Finally, we examined the distribution of MSEL and VABS scores across outcome groups and calculated Spearman correlations comparing the different levels.

Our first set of analyses focused on the association between pre- and postnatal air pollution exposure and adaptive functioning, which was available only for children in the ASD group. We first examined the distribution of fine particulate matter exposure and adaptive skills (VABS). Linear regression models were used to estimate the adjusted beta coefficients and corresponding 95% confidence intervals (CI) for the associations between each 1-unit increase in PM_2.5_ exposure and VABS domains (communication, daily living skills, motor skills, and socialization) and overall VABS composite standardized scores. Next, we assessed associations with pre- and postnatal PM_2.5_ exposure and cognitive functioning among all three SEED groups (ASD, DD, and population controls). Our primary analyses assessed associations with MSEL scores, stratified by outcome group. We additionally assessed associations with all groups combined and adjusted for outcome group (ASD, DD, or POP). Linear regression models were used to estimate betas and 95% CI for the association between PM_2.5_ and MSEL composite and subscale DQs. 

We analyzed associations using trimester-specific exposure averages, as well as exposures averaged over the entire pregnancy period and first year of life. We report mutually adjusted exposure period models [28]. Each trimester-specific result was mutually adjusted for exposure averages during the other trimesters; pregnancy and first year of life models were mutually adjusted for each other. We analyzed PM_2.5_ exposures as continuous measures because a continuous term fit better than categorical coding and because continuous coding allows comparison to previous findings. All results are reported per 1 µg/m^3^ increase in PM_2.5_ exposure. 

A directed acyclic graph was used to identify the minimally sufficient covariate adjustment set (Figure S1). The final DAG-identified adjustment set consisted of the following variables: maternal race/ethnicity (non-Hispanic-white, other race/ethnicity), maternal education at birth (<bachelor’s degree, ≥bachelor’s degree), maternal smoking (any smoking three months before conception or during pregnancy), study site, and year and season of birth. All analyses were conducted using SAS statistical software, version 9.4 (SAS Institute Inc., Cary, NC, USA).

In sensitivity analyses we assessed associations between PM_2.5_ exposure averaged over the pregnancy period and MSEL composite scores, stratified by maternal education and race/ethnicity. For these sensitivity analyses we used a *p*-value of 0.10 to indicate the presence of effect modification.

## 3. Results

### 3.1. Study Population 

The final study sample included 658 children with ASD, 771 with a non-ASD DD, and 849 from the population control group (Table 1). Compared to population controls, children in the ASD or DD groups were more likely to be boys, born preterm, and born to non-white mothers, who had lower than a bachelor’s degree, or who used tobacco during pregnancy. 

PM_2.5_ concentration levels averaged over pregnancy and the first year of life were similar among the groups. Among population controls, correlation coefficients comparing average PM_2.5_ exposures across trimesters ranged from 0.20–0.43, and exposures averaged over the entire pregnancy were strongly correlated (0.92) with exposures in the child’s first year of life (*p* < 0.0001 for all correlations) (Table S1). Correlations were similar for ASD and DD groups. 

### 3.2. Distribution of VABS and MSEL Scores 

The mean MSEL composite score for the POP group was generally comparable to the age-standardized population (i.e., means around 100) (Table S2); however, scores were much lower for the DD and ASD groups. The MSEL composite score was 66.9 among children with ASD, 88.2 in the DD group, and 102.5 in the POP group. MSEL subscale DQ scores were generally highly correlated, particularly for the ASD and DD groups (Table S3). Correlations between the VABS subscale scores (among ASD cases) ranged from 0.45–0.80 (Table S3). 

### 3.3. Associations with VABS Scores 

**Figure 1:**
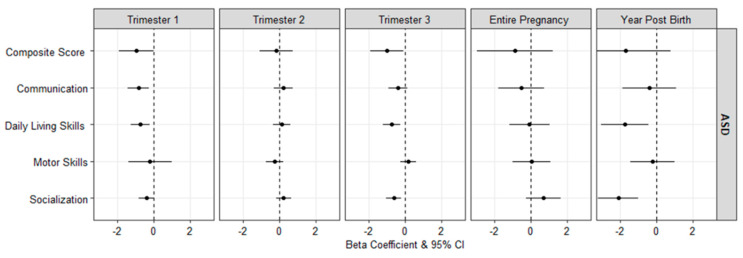
Adjusted mean difference (95% CI) in the scores of the Vineland Adaptive Behavior Scales associated with a 1 µg/m^3^ increase in PM_2.5_, among ASD cases.

Figure 1 presents the adjusted mean differences in VABS scores associated with a 1 µg/m^3^ increase in PM_2.5_ exposure among ASD cases (see Table S4 for numeric results). Among ASD cases, higher concentrations of PM_2.5_ during the first trimester were associated with lower VABS communication and daily living skills scores (β: −0.84, 95% CI: −1.41, −0.27 and β: −0.73, 95% CI: −1.24, −0.21, respectively), and third trimester PM_2.5_ exposure was associated with lower VABS composite scores (β: −0.98, 95% CI: −1.90, −0.06), daily living skills (β: −0.74, 95% CI: −1.23, −0.25), and socialization scores (β: −0.60, 95% CI: −1.02, −0.19). No associations were seen for the second trimester or entire pregnancy period combined. Finally, PM_2.5_ exposure during the first year of life was additionally associated with lower scores on the VABS daily living skills (β: −1.73, 95% CI: −3.04, −0.42) and socialization (β: −2.09, 95% CI: −3.20, −0.97) domains.

### 3.4. Associations with MSEL Scores 

**Figure 2:**
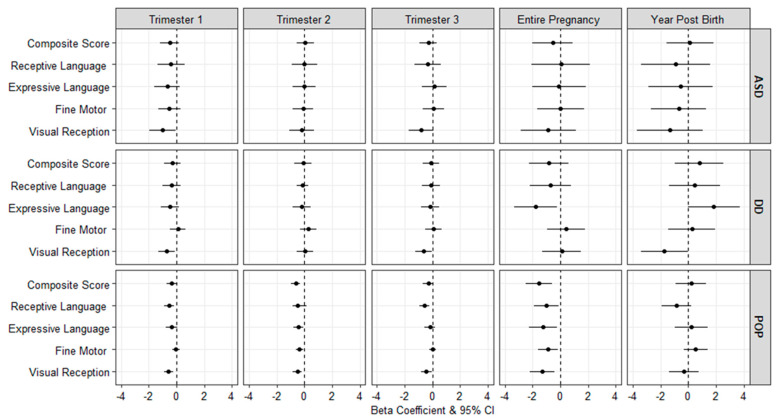
Adjusted mean difference (95% CI) in the scores of the Mullen Scales of Early Learning associated with a 1 µg/m^3^ increase in PM_2.5_ exposure, by outcome classification.

Figure 2 presents adjusted mean differences in the MSEL composite and subscale scores associated with a 1 µg/m^3^ increase in PM_2.5_ for the ASD, DD, and POP groups (see Table S5 for numeric results). Among children in the ASD group, PM_2.5_ exposure during the first trimester was associated with lower scores on the visual reception scale (β: −1.00, 95% CI: −1.94, −0.06. Among children in the DD group, PM_2.5_ exposure during the first and third trimesters and first year of life was associated with lower scores on the MSEL visual reception scale. Additionally, for children in the DD group, PM_2.5_ exposure averaged over pregnancy was associated with lower functioning on the expressive language subscale (β: −1.77, 95% CI: −3.31, −0.23). 

Among the POP group, PM_2.5_ exposure was associated with lower MSEL scores during several of the developmental windows, and most notably when averaged across pregnancy, with lower scores on the MSEL composite score (β: −1.55, 95% CI: −2.48, −0.63), as well as several subscales including receptive language (β: −1.02, 95% CI: −1.92, −0.11), expressive language (β: −1.24, 95% CI: −2.24, −0.24), visual reception (β: −1.33, 95% CI: −2.22, −0.43), and fine motor scores (β: −0.90, 95% CI: −1.62, −0.18) (Figure 2 and Table S5). There were no significant associations with first-year air pollution exposure. 

We additionally assessed associations with MSEL scores with all (ASD, DD, and POP) groups combined (Table S6). In these analyses we observed consistent results between PM_2.5_ exposure and MSEL composite scores for several developmental windows including the first (β: −0.35, 95% CI: −0.65, −0.05) and second (β: −0.27, 95% CI: −0.57, 0.02) trimesters, as well as exposures averaged over the entire pregnancy period (β: −0.97, 95% CI: −1.69, −0.25).

### 3.5. Sensitivity Analyses 

In sensitivity analyses we observed stronger associations between pregnancy average PM_2.5_ exposure and MSEL composite scores for non-Hispanic white (β: −1.86, 95% CI: −2.82, −0.90) compared to non-white (β: −1.11, 95% CI: −2.13, −0.10) children in the POP group (Table S7). Additionally, children in the DD group born to lower educated mothers showed stronger associations with MSEL composite scores (β: −1.28, 95% CI: −2.83, 0.27) compared to children born to higher educated mothers (β: −0.51, 95% CI: −1.99, 0.97).

## 4. Discussion

In this multi-site case-control study, we observed that among children with ASD, higher first trimester PM_2.5_ exposure was associated with poorer scores on the VABS daily living skills domain and MSEL visual reception domain. Higher third trimester and first year PM_2.5_ exposure was associated with poorer scores on the VABS daily living skills and socialization domains. Similar to children with ASD, higher first trimester PM_2.5_ exposure was associated with poorer scores on the MSEL visual reception domain for children in the DD and POP groups. Additionally, among children sampled from the general population, higher prenatal PM_2.5_ exposure was consistently associated with poorer scores on the MSEL visual reception domain regardless of the timing of the exposure within pregnancy and all MSEL domains for the entire pregnancy average. These findings support that prenatal air pollution exposure is associated with specific aspects of adaptive and cognitive functioning in children with and without ASD. Overall, air pollution is a ubiquitous exposure that impacts everyone worldwide; thus, even small differences in functioning may result in a considerable public health burden. 

Our current results are in agreement with other epidemiologic findings from the US and abroad that have implicated third trimester and first year of life air pollution exposure (including PM_2.5_ and nitrogen dioxide (NO_2_)) as windows of susceptibility for ASD risk [11,12,14,29,30]. Specifically relating to adaptive deficits, both Kerin et al. [15] and the current study found that first year of life air pollution exposure was associated with lower scores on the VABS daily living skills and socialization domains. Additionally, we found that third trimester PM_2.5_ exposure was also associated with lower scores on the VABS daily living skills and socialization domains. Collectively, these findings suggest that air pollution exposure in late pregnancy and early life may negatively impact social and independent skill development among children with ASD. 

We assessed impacts with PM_2.5_ exposure and MSEL scores stratified by outcome group and in pooled analyses adjusted for outcome group. In stratified analyses, the most robust association between PM_2.5_ and cognitive function was with the visual reception domain. Further, the POP group showed significant associations between PM_2.5_ exposure averaged over the entire pregnancy period and other developmental windows including the second trimester and lower visual reception, expressive language, receptive language, and fine motor scores. These findings are in line with a few recent studies on early life air pollution exposure and cognitive domains in children. Specifically, associations have been found in recent studies for second trimester air pollution exposure and cognitive and communicative scores in children [18]. Additionally, other studies have found impacts from prenatal air pollution exposure and deficits in fine motor skills [17] and general facets of behavioral development [19].

Several specific mechanisms may link early life air pollution exposure to deficits in children’s adaptive behavior and cognitive development [31]. The structures and critical processes of the brain—such as visual reception—begin to develop in the first trimester and continue to develop rapidly throughout the neonatal period. Some of these processes include neuron formation and migration, generation of glial cells, and myelination [32]. There is an extensive body of toxicological literature linking maternal particulate matter exposure to inflammation, oxidative stress, and the production of pro-inflammatory cytokines [33]. These pro-inflammatory cytokines may reach the developing brain, resulting in neuroinflammation, neuron damage/loss, and microglia activation [32]. Further migration, differentiation, and synaptic pruning continues after birth and through early childhood [34]. Disruptions of these critical processes could alter normal postnatal brain development and compound social and independent skill deficits seen in children with ASD. Thus, the timing of exposure to air pollution may produce differential risks of adaptive and cognitive impairments evident later in childhood and throughout the life course. 

The strengths of this study include the use of gold standard outcome assessment tools for ASD ascertainment; the assessment of associations with cognitive function in three different diagnostic groups; the use of high quality PM_2.5_ exposure estimates generated from a state-of-the-art satellite-based exposure model; and the use of mutual adjustment models to control for confounding by correlated windows of exposure. Our study is not without limitations. We adjusted for race, ethnicity, and maternal education in all analyses; however, we cannot exclude the possibility of residual confounding by socioeconomic status or other factors. Like other air pollution epidemiology studies, we used an area level air pollution measure at birth as a proxy for individual-level exposure for the entire pregnancy period and the year post birth. Previous studies have shown little change in exposure ranking and identified the critical window when incorporating the full residential history [35,36]. It is also possible that mothers could have worked in areas with different air pollution levels. We did not have information on location of employment during pregnancy and note this as a limitation. Further, SEED maintained confidentiality of participants by shifting all dates by up to two weeks in either direction. We conducted sensitivity analyses in the one site for which both actual and shifted data were available and found that the shifting did not impact trimester, pregnancy, or first year of life (i.e., longer exposure) averaging periods. This shifting prevented us from confidently studying impacts from shorter exposure periods such as weeks, and we acknowledge this as a limitation. 

## 5. Conclusions

We assessed the association between early life PM_2.5_ exposure and measures of both adaptive behavior and cognitive development in young children. These findings expand upon other epidemiologic studies that have found associations between early life air pollution exposure and adaptive and cognitive functioning in children [37,38,39] by delineating specific developmental domains among children with and without ASD. The role air pollutants play in neurodevelopment and the ability to detect relevant associations may differ depending on the prominence of other factors that influence neurodevelopment among sub-groups of children. Additional studies, including animal studies, could increase the understanding of how specific components of air pollution and timing of exposure are related to child outcomes, particularly visual reception or nonverbal learning skills. 

## Figures and Tables

**Table 1:** Characteristics [*n* (%) or mean ± SD] of the Study to Explore Early Development population by outcome classification.

Characteristic	ASD (*n* = 658)	DD (*n* = 771)	POP (*n* = 849)	ASD vs. POP *p*-Value	DD vs. POP *p*-Value
Child sex					
Male	537 (82)	492 (64)	449 (53)		
Female	121 (18)	279 (36)	400 (47)	<0.0001	<0.0001
Birth Year					
2003–2004	267 (41)	463 (60)	458 (54)		
2005–2006	391 (59)	308 (40)	391 (46)	0.02	0.01
Maternal race/ethnicity					
Non-Hispanic White	367 (56)	478 (62)	608 (72)		
Other ^a^	291 (44)	293 (38)	241 (28)	<0.0001	<0.0001
Maternal education					
<Bachelor’s	322 (49)	335 (43)	286 (34)		
≥Bachelor’s	336 (51)	436 (57)	563 (66)	<0.0001	0.0002
Maternal age at birth (years)					
<35	471 (72)	538 (70)	587 (69)		
≥35	187 (28)	233 (30)	262 (31)	0.20	0.79
Tobacco use during pregnancy					
Yes	106 (16)	106 (14)	78 (9)		
No	552 (84)	665 (86)	771 (91)	<0.0001	0.004
Preterm					
Yes	109 (17)	172 (22)	80 (9)		
No	549 (83)	599 (78)	769 (91)	<0.0001	<0.0001
PM_2.5_ (µg/m^3^) (mean ± SD)					
Pregnancy	12.8 ± 2.7	12.9 ± 2.6	12.7 ± 2.6	0.37	0.18
First year of life	12.7 ± 2.5	12.8 ± 2.5	12.5 ± 2.5	0.12	0.10

Numbers are N (%) or mean ± SD. ASD indicates autism spectrum disorder; DD, non-ASD developmental delays or disorders; PM_2.5_, particulate matter < 2.5 µm; POP, population-based control group; SD, standard deviation. ^a^ Includes African American, Asian, Hispanic, and all other.

## Data Availability

A minimal dataset may be available upon request. The data are not publicly available due to privacy restrictions.

## Supplementary Materials

The following supporting information can be downloaded at: https://www.mdpi.com/article/10.3390/ijerph19073748/s1, Figure S1: Directed Acyclic Graph of the relationship between air pollution exposure and cognitive and adaptive function; Table S1: Spearman correlation coefficients for modeled PM_2.5_ (µg/m^3^) estimates averaged across developmental windows; Table S2: Distribution of Vineland Adaptive Behavior Scales and Mullen Scales of Early Learning scores by outcome classification; Table S3: Spearman correlations between MSEL and VABS scores, by outcome classification group; Table S4: Adjusted mean difference (95% CI) in the scores of the Vineland Adaptive Behavior Scales associated with a 1 µg/m^3^ increase in PM_2.5_ exposure, among ASD cases only; Table S5: Adjusted mean difference (95% CI) in the scores of the Mullen Scales of Early Learning associated with a 1 µg/m^3^ increase in PM_2.5_ exposure, stratified by outcome classification; Table S6: Adjusted mean difference (95% CI) in the scores of the Mullen Scales of Early Learning associated with a 1 µg/m^3^ increase in PM_2.5_ exposure, adjusted for outcome classification. Table S7: Adjusted mean difference (95% CI) in the Mullen Scales of Early Learning Composite Score associated with a 1 µg/m^3^ increase in PM_2.5_ exposure averaged across the pregnancy period, by outcome classification. Results are stratified by maternal race and education.
