# Supplementary material for: Pre- and Postnatal Fine Particulate Matter Exposure and Childhood Cognitive and Adaptive Function
Source: Int J Environ Res Public Health. 2022 Mar 22;19(7):3748. doi: 10.3390/ijerph19073748 (PMC8997879; doi:10.3390/ijerph19073748)
Supplement: Supplementary file 1 [file ijerph-19-03748-s001.zip › ijerph-1605729-supplementary.pdf]

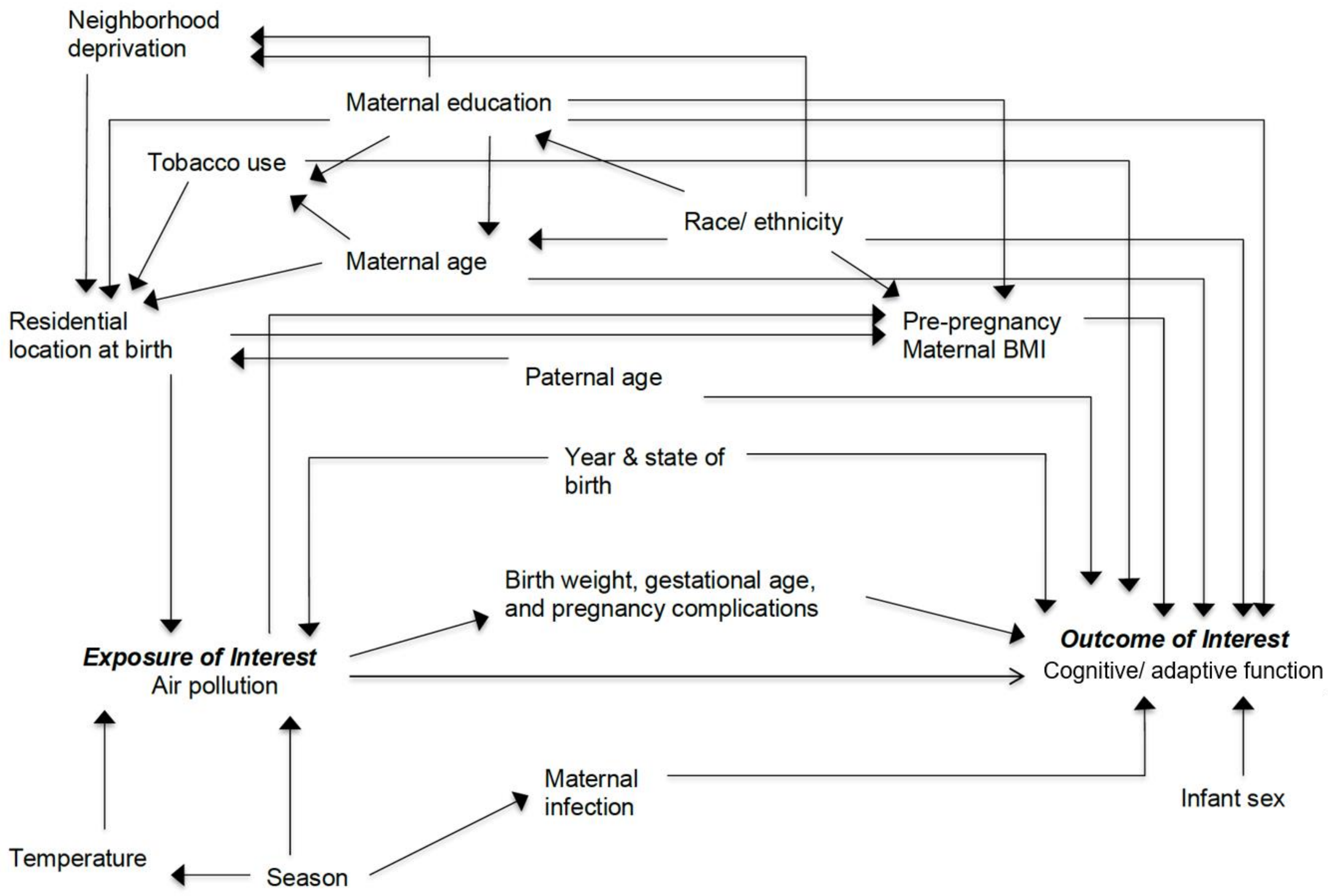

Figure S1. Directed Acyclic Graph of the relationship between air pollution exposure and cognitive and adaptive function.

Table S1. Spearman correlation coefficients[a] for modeled $PM_{2.5}$ (μg/m^3) estimates averaged across developmental windows

(a) ASD

	First trimester	Second trimester	Third trimester	Entire Pregnancy	1st year
First trimester	1.0				
Second trimester	0.44	1.0			
Third trimester	0.26	0.44	1.0		
Entire pregnancy	0.74	0.82	0.73	1.0	1.0
1st year	0.70	0.68	0.70	0.91	1.0

(b) DD

	First trimester	Second trimester	Third trimester	Entire Pregnancy	1st year
First trimester	1.0				
Second trimester	0.35	1.0			
Third trimester	0.19	0.43	1.0		
Entire pregnancy	0.69	0.80	0.72	1.0	1.0
1st year	0.65	0.67	0.70	0.90	1.0

(c) controls

	First trimester	Second trimester	Third trimester	Entire Pregnancy	1st year
First trimester	1.0				
Second trimester	0.35	1.0			
Third trimester	0.18	0.42	1.0		
Entire pregnancy	0.68	0.80	0.72	1.0	1.0
1st year	0.66	0.66	0.70	0.92	1.0

$PM_{2.5}$ indicates particulate matter <2.5 μm.

[a] $p < 0.0001$ for all values

Table S2. Distribution of Vineland Adaptive Behavior Scales[a] and Mullen Scales of Early Learning scores by outcome classification[b].

	ASD					DD					POP				
	N	Mean	Percentiles			N	Mean	Percentiles			N	Mean	Percentiles		
			5	50	95			5	50	95			5	50	95
**Mullen (DQ scores)**															
Composite score	658	66.9	49	63	108	771	88.2	49	91	118	849	102.5	78	103	125
Receptive language	658	63.4	16	63	108	771	90.6	49	95	121	849	103.9	82	104	125
Expressive language	658	59.7	13	63	102	771	86.5	43	89	119	849	101.2	74	103	125
Fine motor	658	68.0	30	70	103	771	88.7	48	94	113	849	99.9	82	100	116
Visual reception	658	73.8	30	76	117	771	94.3	53	98	120	849	103.5	83	103	125
**Vineland Adaptive Behavior Scales (standard scores)**															
Composite score	658	75.7	50	74	98										
Communication	658	77.0	42	79	104										
Daily living skills	658	74.5	48	75	100										
Motor skills	658	80.1	56	80	104										
Socialization	658	72.6	53	72	95										

ASD indicates autism spectrum disorder; DD, non-ASD developmental delays or disorders; POP, population-based control group.

[a] Vineland scores are only available for the ASD group

[b] Mean values are all significantly ($p < 0.0001$) different from the population mean value of 100, except for the population control group's fine motor and expressive language means.

Table S3. Spearman correlations[a] between MSEL and VABS scores, by outcome classification group.

	MSEL Composite	MSEL Receptive Language	MSEL Expressive Language	MSEL Fine Motor	MSEL Visual Reception	VABS Composite	VABS Commun -ication	VABS Daily Living	VABS Motor Skills	VABS Social-ization
**ASD**										
MSEL composite	1.00									
MSEL receptive language	0.93	1.00								
MSEL expressive language	0.89	0.86	1.00							
MSEL fine motor	0.84	0.72	0.65	1.00						
MSEL visual reception	0.87	0.78	0.70	0.75	1.00					
VABS composite	0.30	0.28	0.27	0.36	0.32	1.00				
VABS communication	0.68	0.66	0.64	0.62	0.67	0.46	1.00			
VABS daily living	0.56	0.54	0.52	0.54	0.57	0.51	0.80	1.00		
VABS motor skills	0.51	0.46	0.41	0.58	0.51	0.43	0.68	0.74	1.00	
VABS socialization	0.54	0.50	0.59	0.48	0.58	0.45	0.73	0.78	0.64	1.00
**DD**										
MSEL composite	1.00									
MSEL receptive language	0.92	1.00								
MSEL expressive language	0.89	0.83	1.00							
MSEL fine motor	0.84	0.69	0.65	1.00						
MSEL visual reception	0.85	0.73	0.64	0.66	1.00					
**POP**										
MSEL composite	1.00									
MSEL receptive language	0.86	1.00								
MSEL expressive language	0.79	0.66	1.00							
MSEL fine motor	0.73	0.48	0.42	1.00						
MSEL visual reception	0.74	0.53	0.37	0.44	1.00					

ASD indicates autism spectrum disorder; DD, non-ASD developmental delays or disorders; MSEL, Mullen Scales of Early Living; POP, population-based control group; VABS, Vineland Adaptive Behavior Scales.

[a] $p < 0.0001$ for all values

Table S4. Adjusted mean difference (95% CI) in the scores of the Vineland Adaptive Behavior Scales associated with a 1-µg/m^3 increase in $PM_{2.5}$ exposure, among ASD cases only.

	First Trimester	Second Trimester	Third Trimester	Entire Pregnancy	First Year
Composite score	−0.95 (−1.92, 0.02)	−0.17 (−1.06, 0.73)	−0.98 (−1.90, −0.06)	−0.85 (−2.95, 1.25)	−1.69 (−4.15, 0.78)
Communication	−0.84 (−1.41, −0.27)	0.23 (−0.30, 0.76)	−0.38 (−0.92, 0.16)	−0.52 (−1.77, 0.74)	−0.38 (−1.86, 1.09)
Daily living skills	−0.73 (−1.24, −0.21)	0.12 (−0.35, 0.60)	−0.74 (−1.23, −0.25)	−0.06 (−1.17, 1.05)	−1.73 (−3.04, −0.42)
Motor skills	−0.19 (−1.39, 1.01)	−0.25 (−0.73, 0.22)	0.17 (−0.27, 0.61)	0.05 (−0.98, 1.09)	−0.19 (−1.41, 1.03)
Socialization	−0.38 (−0.82, 0.05)	0.24 (−0.16, 0.65)	−0.60 (−1.02, −0.19)	0.72 (−0.23, 1.66)	−2.09 (−3.20, −0.97)

Adjusted for maternal age, maternal education, tobacco use during pregnancy, sex, child race/ethnicity, site, and season of birth

ASD indicates autism spectrum disorder; CI, confidence interval; $PM_{2.5}$, particulate matter <2.5 µm.

Table S5. Adjusted mean difference (95% CI) in the scores of the Mullen Scales of Early Learning associated with a 1-µg/m^3 increase in $PM_{2.5}$ exposure, stratified by outcome classification.

	**First Trimester**	**Second Trimester**	**Third Trimester**	**Entire Pregnancy**	**First Year**
**ASD**					
Composite score	−0.49 (−1.17, 0.18)	0.05 (−0.58, 0.67)	−0.30 (−0.93, 0.34)	−0.57 (−2.01, 0.87)	0.11 (−1.60, 1.81)
Receptive language	−0.40 (−1.39, 0.60)	−0.02 (−0.94, 0.90)	−0.33 (−1.29, 0.62)	0.05 (−2.07, 2.16)	−0.93 (−3.44, 1.58)
Expressive language	−0.67 (−1.58, 0.24)	−0.02 (−0.86, 0.82)	0.13 (−0.74, 1.00)	−0.10 (−2.04, 1.84)	−0.56 (−2.86, 1.74)
Fine motor	−0.51 (−1.30, 0.28)	−0.11 (−0.84, 0.62)	0.06 (−0.69, 0.82)	0.02 (−1.67, 1.70)	−0.70 (−2.69, 1.30)
Visual reception	−1.00 (−1.94, −0.06)	−0.20 (−1.07, 0.67)	−0.83 (−1.73, 0.07)	−0.87 (−2.88, 1.14)	−1.33 (−3.70, 1.04)
DD					
Composite score	−0.30 (−0.90, 0.31)	−0.09 (−0.71, 0.52)	−0.11 (−0.72, 0.51)	−0.83 (−2.25, 0.60)	0.78 (−0.95, 2.51)
Receptive language	−0.36 (−1.00, 0.27)	−0.12 (−0.76, 0.52)	−0.12 (−0.76, 0.52)	−0.75 (−2.23, 0.74)	0.45 (−1.37, 2.26)
Expressive language	−0.49 (−1.14, 0.16)	−0.19 (−0.84, 0.48)	−0.16 (−0.82, 0.50)	−1.77 (−3.31, −0.23)	1.84 (−0.03, 3.72)
Fine motor	0.09 (−0.49, 0.68)	0.26 (−0.33, 0.86)	0.05 (−0.54, 0.64)	0.41 (−0.98, 1.79)	0.25 (−1.44, 1.93)
Visual reception	−0.71 (−1.30, −0.12)	0.02 (−0.58, 0.62)	−0.66 (−1.25, −0.07)	0.09 (−1.29, 1.47)	−1.73 (−3.41, −0.04)
POP					
Composite score	−0.35 (−0.71, 0.01)	−0.64 (−0.98, −0.30)	−0.30 (−0.68, 0.07)	−1.55 (−2.48, −0.63)	0.19 (−0.91, 1.29)
Receptive language	−0.51 (−0.87, −0.16)	−0.52 (−0.85, 0.18)	−0.59 (−0.96, −0.22)	−1.02 (−1.92, −0.11)	−0.88 (−1.94, 0.19)
Expressive language	−0.36 (−0.75, 0.03)	−0.45 (−0.81, −0.08)	−0.19 (−0.59, 0.22)	−1.24 (−2.24, −0.24)	0.21 (−0.97, 1.39)
Fine motor	−0.04 (−0.32, 0.24)	−0.36 (−0.63, −0.10)	0.001 (−0.29, 0.30)	−0.90 (−1.62, −0.18)	0.52 (−0.33, 1.37)
Visual reception	−0.57 (−0.92, −0.22)	−0.52 (−0.85, −0.19)	−0.44 (−0.80, −0.07)	−1.33 (−2.22, −0.43)	−0.34 (−1.39, 0.72)

Adjusted for maternal age, maternal education, tobacco use during pregnancy, sex, child race/ethnicity, site, and season of birth

ASD indicates autism spectrum disorder; CI, confidence interval; DD, non-ASD developmental delays or disorders; $PM_{2.5}$, particulate matter <2.5 µm; POP, population-based control group.

Table S6. Adjusted mean difference (95% CI) in the scores of the Mullen Scales of Early Learning associated with a 1-µg/m^3 increase in $PM_{2.5}$ exposure, adjusted for outcome classification.

	First Trimester	Second Trimester	Third Trimester	Entire Pregnancy	First Year
Composite score	−0.35 (−0.65, −0.05)	−0.27 (−0.57, 0.02)	−0.18 (−0.49, 0.12)	−0.97 (−1.69, −0.25)	0.38 (−0.47, 1.23)
Receptive language	−0.26 (−0.89, 0.38)	−0.37 (−0.99, 0.24)	−0.11 (−0.75, 0.53)	0.01 (−1.48, 1.50)	−1.32 (−3.10, 0.45)
Expressive language	−0.47 (−0.83, −0.12)	−0.24 (−0.59, 0.10)	−0.04 (−0.40, 0.32)	−1.11 (−1.95, −0.26)	0.61 (−0.40, 1.61)
Fine motor	0.02 (−0.60, 0.63)	−0.21 (−0.81, 0.39)	0.26 (−0.36, 0.89)	0.48 (−0.98, 1.93)	−0.91 (−2.64, 0.82)
Visual reception	−0.55 (−1.35, 0.24)	−0.39 (−1.16, 0.38)	−0.34 (−1.15, 0.46)	0.17 (−1.69, 2.04)	−2.37 (−4.59, −0.15)

Adjusted for maternal age, maternal education, tobacco use during pregnancy, sex, child race/ethnicity, site, and season of birth

ASD indicates autism spectrum disorder; CI, confidence interval; DD, non-ASD developmental delays or disorders; $PM_{2.5}$, particulate matter <2.5 µm; POP, population-based control group.

Table S7. Adjusted mean difference (95% CI) in the Mullen Scales of Early Learning Composite Score associated with a 1-μg/m^3 increase in $PM_{2.5}$ exposure averaged across the pregnancy period, by outcome classification. Results are stratified by maternal race and education.

	**Non−Hispanic White**	**Other Race/Ethnicity**	***p−int***	**<Bachelor's**	**≥Bachelor's**	***p−int***
ASD	−0.91 (−2.46, 0.63)	−0.30 (−1.80, 1.19)	0.28	−0.61 (−2.11, 0.88)	−0.52 (−2.05, 1.01)	0.87
DD	−0.84 (−2.31, 0.64)	−0.84 (−2.41, 0.73)	0.99	−1.28 (−2.83, 0.27)	−0.51 (−1.99, 0.97)	0.19
POP	−1.86 (−2.82, −0.90)	−1.11 (−2.13, −0.10)	0.05	−1.47 (−2.47, −0.46)	−1.61 (−2.58, −0.65)	0.68
